# Possible Protective Effect of Diacerein on Doxorubicin-Induced Nephrotoxicity in Rats

**DOI:** 10.1155/2016/9507563

**Published:** 2016-01-20

**Authors:** Marwa M. M. Refaie, Entesar F. Amin, Nashwa F. El-Tahawy, Aly M. Abdelrahman

**Affiliations:** ^1^Department of Pharmacology, Faculty of Medicine, El-Minia University, El-Minia 61111, Egypt; ^2^Department of Histology, Faculty of Medicine, El-Minia University, El-Minia 61111, Egypt

## Abstract

Nephrotoxicity is one of the limiting factors for using doxorubicin (DOX). Interleukin 1 has major role in DOX-induced nephrotoxicity, so we investigated the effect of interleukin 1 receptor antagonist diacerein (DIA) on DOX-induced nephrotoxicity. DIA (25 and 50 mg/kg/day) was administered orally to rats for 15 days, in the presence or absence of nephrotoxicity induced by a single intraperitoneal injection of DOX (15 mg/kg) at the 11th day. We measured levels of serum urea, creatinine, renal reduced glutathione (GSH), malondialdehyde (MDA), total nitrites (NO_*x*_), catalase, and superoxide dismutase (SOD). In addition, caspase-3, tumor necrosis factor alpha (TNF*α*), nuclear factor kappa B (NF*κ*B) expressions, and renal histopathology were assessed. Our results showed that DOX-induced nephrotoxicity was ameliorated or reduced by both doses of DIA, but diacerein high dose (DHD) showed more improvement than diacerein low dose (DLD). This protective effect was manifested by significant improvement in all measured parameters compared to DOX treated group by using DHD. DLD showed significant improvement of creatinine, MDA, NO_*x*_, GSH, histopathology, and immunohistochemical parameters compared to DOX treated group.

## 1. Introduction 

Drug-induced nephrotoxicity is a major cause of acute kidney injury [1]. DOX is one of the key chemotherapeutic drugs for cancer treatment, but its use is limited by chronic and acute toxic side effects [2]. DOX is an antibiotic anthracycline that was isolated from a pigment of* Streptomyces peucetius* in the early 1960s and it had been employed for more than 30 years in the battle against cancer, but it is now chemically synthesized [3]. The exact mechanism of DOX-induced nephrotoxicity is not yet completely understood. Renal DOX-induced toxicity may be part of a multiorgan damage mediated mainly through free radical formation eventually leading to membrane lipid peroxidation [4]. Induction of apoptosis and modulation of NO_*x*_ are mechanisms that are involved in toxic adverse effects associated with DOX therapy [5]. In addition, DOX has a direct renal damaging effect as it accumulates preferentially in the kidney. DOX has toxic effects on other organs such as heart and liver which may lead to modulation of blood supply to the kidney and alter xenobiotic detoxification processes, respectively, thus indirectly contributing to DOX-induced nephropathy [6].

DIA is a new anti-inflammatory, analgesic, and antipyretic drug that was developed specially for the treatment of osteoarthritis [7]. It is highly effective in relieving the symptoms of osteoarthritis and may be able to modify the course of the disease [7]. DIA acts by inhibiting the production of interleukin 1 by human monocytes [8]. Interleukin 1 is a proinflammatory and proapoptotic agent that induces cytokine production by activating NF*κ*B and mitogen activated protein kinase signaling [9]. A major cause of DOX-induced nephrotoxicity is the production of reactive oxygen species which induce cytokines, including interleukin 1 [6, 9, 10]. The aim of the present study was to study the effect of the interleukin 1 receptor antagonist diacerein (DIA) on DOX-induced nephropathy.

## 2. Materials and Methods

### 2.1. Chemicals

DIA powder was from Eva Pharma Company and it was dissolved in 1% carboxymethylcellulose. DOX hydrochloride 10 mg vial (Pharmacia Italia, SPA, Italy), polyclonal rabbit/antirat caspase-3, TNF*α*, and NF*κ*B antibody (Lab Vision, USA), biotinylated goat anti-rabbit secondary antibody (Transduction Laboratories, USA), urea, GSH, SOD, and catalase kits (Biodiagnostic, Egypt), and creatinine (Humen, Germany) were purchased.

### 2.2. Animals

Adult male Wistar rats weighing about 250–350 g were obtained from the Animal Research Centre, Giza, Egypt. Animals were kept in standard housing conditions in cages and were left to acclimatize for one week. Rats were supplied with laboratory chow and tap water. This work was conducted in the Pharmacology Department, Faculty of Medicine, El-Minia University, Egypt, and the animal experimental protocol was approved by the faculty board.

### 2.3. Experimental Design

Rats were randomly assigned into 6 groups (*n* = 6 each) as follows.

 Group I received vehicle (1% carboxymethylcellulose) for 15 days and ip saline at day 11.
 Group II was treated with DLD (25 mg/kg/d orally) for 15 days and ip saline at day 11.
 Group III was treated with DHD (50 mg/kg/d orally) and ip saline at day 11.
 Group IV was treated with vehicle for 15 days and DOX (15 mg/kg) at day 11.
 Group V was treated with DLD (25 mg/kg/d orally) for 15 days + ip injection of DOX (15 mg/kg) at day 11.
 Group VI was treated with DHD (50 mg/kg/d orally) for 15 days + ip injection of DOX (15 mg/kg) at day 11. The doses of DOX and DIA were based on the previous studies [4, 11].

### 2.4. Evaluation of Renal Function

After 4 days of DOX injection, each rat was weighed then anesthetized with ip injection of urethane (25% in a dose of 1.6 gm/kg) and then sacrificed.

Venous blood samples were collected from the jugular vein.

Serum was collected for biochemical analysis of urea [12] and creatinine [13]. They were determined using colorimetric diagnostic kits according to the manufacturer's instructions.

After sacrifice, both kidneys were rapidly excised and weighed.

A longitudinal section of the left kidney and one half was fixed in 10% formalin then embedded in paraffin for histopathological and immunohistochemical examinations. The rest of the kidneys were snap frozen in liquid nitrogen and kept at −80°C.

### 2.5. Evaluation of GSH

GSH spectrophotometric kit was used. Briefly, the method is based on the fact that sulfhydryl group of GSH reacts with 5, 5′-dithiobis (2-nitrobenzoic acid) (Ellman's reagent) and produces a yellow colored 5-thio-2-nitrobenzoic acid which was measured colorimetrically at 405 nm using Beckman DU-64 UV/VIS spectrophotometer, USA. Results were expressed as mmol/g tissue [14].

### 2.6. Evaluation of Renal Catalase Levels

Assessment of renal catalase antioxidant enzyme activity was determined from the rate of decomposition of H_2_O_2_ at 510 nm after the addition of tissue homogenate as described by colorimetric kit. The results were expressed as unit/g tissue [15].

### 2.7. Evaluation of SOD Levels

The assessments of SOD levels were based on the ability of the enzyme to inhibit the phenazine methosulfate-mediated reduction of nitroblue tetrazolium dye and the results were expressed as unit/g tissue [16].

### 2.8. Assessment of Renal Lipid Peroxides

#### 2.8.1. Principle

The renal contents of lipid peroxides were assayed by a spectrophotometric method based on the reaction between MDA and thiobarbituric acid [17].

#### 2.8.2. Procedure

The absorbance values of the samples and the blank were determined at 535 nm using a (Beckman DU-64 spectrophotometer, USA) and then blank absorbance value was subtracted from the sample absorbance value. From a standard curve, MDA concentration in the unknown sample was extrapolated from the corresponding absorbance using the regression line from the standard curve and expressed as nmol/gm tissue by multiplying in the tissue dilution factor.

### 2.9. Assessment of NO_*x*_ Levels

#### 2.9.1. Principle

Nitric oxide (NO) in the form of nitrite was determined with spectrophotometric method using Griess reagent systems. The stable oxidation end products of NO, nitrite (NO_2_^−^), and nitrate (NO_3_^−^) were used as indicators of NO production. NO_*x*_ was measured after the reduction of nitrate to nitrite by copperized cadmium granules in glycine buffer at pH 9.7. Quantification of NO_2_^−^ was based on the Griess reaction, in which a chromophore with a strong absorbance at 540 nm is formed by the reaction of nitrite with a mixture of N-naphthylene diamine and sulfanilamide [18]. The absorbance of the sample and the blank were measured at 545 nm using (Beckman DU-64 spectrophotometer, USA). The blank absorbance is then subtracted from the sample absorbance.

From a standard curve, NO_*x*_ content in the unknown sample was extrapolated from the corresponding absorbance using the regression line from the standard curve and expressed as nmol/g tissue.

### 2.10. Histological Examination

Renal tissue was fixed in 10% formalin, embedded in paraffin, sectioned by a microtome at 5 *μ*m thickness and stained with hematoxylin and eosin for routine histopathological assessment.

#### 2.10.1. Morphometric Study

The renal tissues were examined in random microscopic areas semiquantitatively under 40 high power fields and the number of changes was assessed by the counting of 3 nonoverlapped fields for the same slide of each animal. The frequency and the severity of lesions in the kidneys were assessed semiquantitatively as follows: Score −: assigned normal, Score +: in between normal and mild, Score ++ (mild level): less than 25% of the examined fields revealed histological alterations, Score +++ (moderate level): less than 50% of the examined fields revealed histological alterations, and Score ++++ (severe level): less than 75% of the total fields examined revealed histological alterations [19].

### 2.11. Immunohistochemical Examination

The caspase-3, TNF*α*, and NF*κ*B immunolabeled cells were counted. In each animal, 3 sections were examined and the cells were counted in 3 adjacent nonoverlapping fields levels. Immunohistochemical staining was performed for caspase-3, TNF*α*, and NF*κ*B using polyclonal rabbit/antirat antibody according to previously published protocol [20, 21], respectively.

### 2.12. Statistical Analysis

Data was analyzed by one way ANOVA followed by Dunnett multiple comparison test. The values are represented as means ± SEM. Statistical analysis was done using GraphPad Prism software (version 5). The differences were considered significant when the calculated *P* value is less than 0.05.

## 3. Results

### 3.1. Effect of DIA on Urea and Creatinine in DOX Treated Rats

Table 1 shows the results of the effect of DIA on serum creatinine and urea. Rats receiving a single dose of DOX (15 mg/kg, ip) showed a significant increase in serum creatinine and urea levels compared to control group. Both doses of DIA resulted in significant decrease in serum creatinine compared to DOX treated rats. DIA 50 mg/kg/day but not 25 mg/kg/day resulted in significant decrease in serum urea compared to DOX treated rats.

### 3.2. Effect of DIA on MDA and NO_*x*_ Levels in DOX-Induced Nephrotoxicity

Renal MDA was evaluated as an indicator of kidney lipid peroxidation and nitrites and nitrates as an indicator of renal NO_*x*_ levels (Table 1). DOX (15 mg/kg) significantly increased renal MDA and NO_*x*_ levels compared to control group. Administrating both doses of DIA to DOX treated rats significantly decreased MDA and NO_*x*_ compared to DOX treated group.

### 3.3. Effect of DIA on GSH, SOD, and Catalase Levels in DOX-Induced Nephrotoxicity

Treatment with DOX (15 mg/kg) caused significant decrease in renal GSH, SOD, and catalase levels compared with untreated control group (Table 2). Concomitant treatment of DOX with DIA significantly increased the levels of renal GSH, SOD, and catalase compared to DOX treated group.

### 3.4. Histological Results

**Figure 1:**
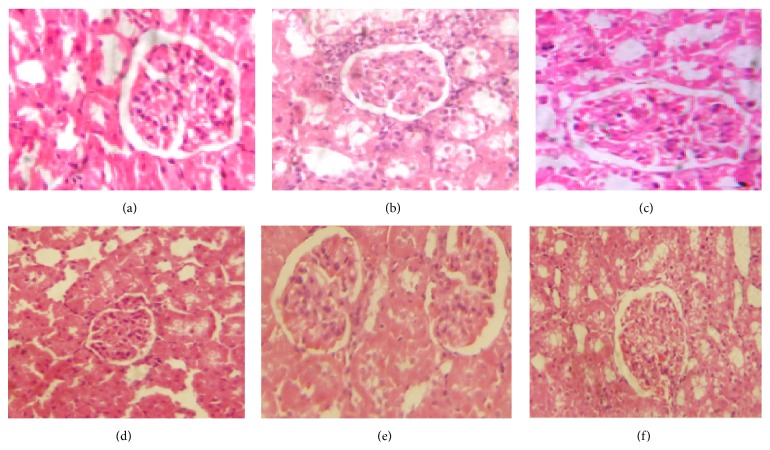
Photomicrographs of renal cortex of (a), (b), and (c), control, DLD, and DHD groups, respectively, showing normal lobular organization of the renal cortex; normal renal glomeruli and tubules. (d) DOX treated group showing markedly enlarged and congested vascular renal glomeruli and cytoplasmic vacuolations of corpuscular cells. Inflammatory cell infiltrations are observed. (e) DOX/DLD group showing less cytoplasmic vacuolations of the renal corpuscular cells and tubular cells. (f) DOX/DHD showing apparent normal renal cortex. H&E ×400. Bar = 20 *μ*.

The histological study of the rat renal cortical tissue of control group (Figure 1(a)), DLD (25 mg/kg/day) group (Figure 1(b)), and DHD (50 mg/kg/day) group (Figure 1(c)) showed normal architecture of renal glomeruli and tubules. DOX treated group (Figure 1(d)) showed marked enlargement of some vascular glomeruli which tightly fill the renal corpuscles. Most renal corpuscular and tubular cells showed abundant cytoplasmic vacuolations and tubular distortion. Interstitial inflammatory cells infiltrations were observed. DOX + DLD group (Figure 1(e)) showed amelioration of the damaging effects of DOX. There were less tubular distortion, narrow Bowman's spaces, and fewer cytoplasmic vacuolations of renal corpuscle and tubular cells were also observed. DOX + DHD group (Figure 1(f)) had more obvious decrease in the morphological changes caused by DOX exposure.

### 3.5. Morphometric Results

The severity of the morphological changes was assessed semiquantitatively; DOX exposed group showed increase in the glomerular and tubular morphological changes at the light microscopic levels when compared with control group. These changes were suppressed by the administration of both doses of DIA, but the high dose showed marked improvement than the low dose (Table 3).

### 3.6. Immune-Histochemical Results

**Figure 2:**
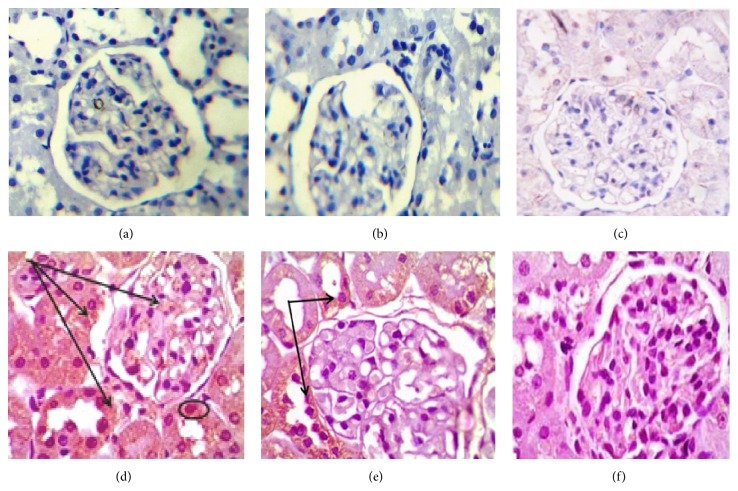
Photomicrographs of renal cortex immune stained for caspase-3 of (a), (b), and (c), control, DLD, and DHD groups, respectively, showing negative immunoreactivity. (d) DOX treated group showing extensive expression in the renal glomeruli and renal tubules. (e) DOX/DLD group showing moderate expression within the glomeruli and the renal tubules. (f) DOX/DHD group showed marked improvement with no expression in glomeruli and renal tubules. The expression is mainly cytoplasmic, but with some immunopositive nuclei. Immunohistochemistry counter stained with H&E ×400. Bar = 20 *μ*.

**Figure 3:**
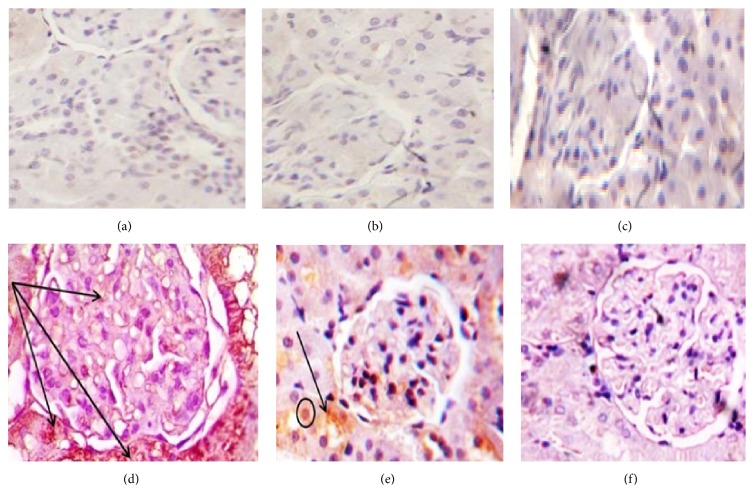
Photomicrographs of renal cortex immune stained for NF*κ*B of (a), (b), and (c), control, DLD, and DHD groups, respectively, showing negative immunoreactivity. (d) DOX treated group showing extensive expression in the renal glomeruli and renal tubules. (e) DOX/DLD group showing moderate expression within the glomeruli and the renal tubules. (f) DOX/DHD group showed marked improvement with no expression in glomeruli and renal tubules. The expression is mainly cytoplasmic but with some immunopositive nuclei. Immunohistochemistry counter stained with H&E ×400. Bar = 20 *μ*.

**Figure 4:**
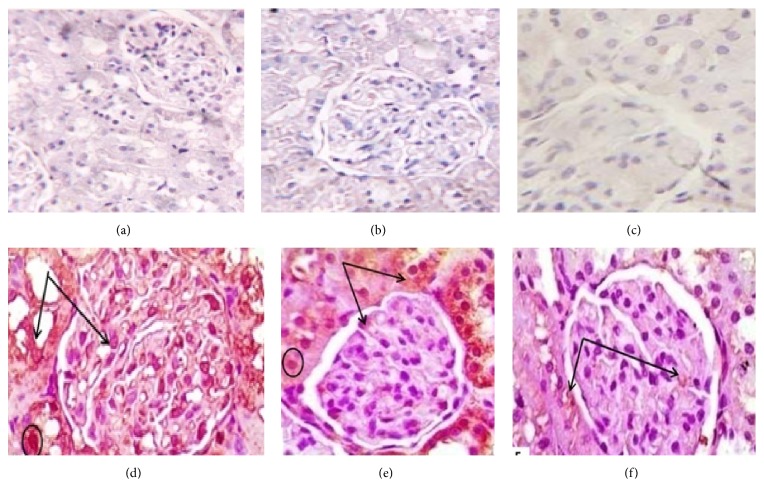
Photomicrographs of renal cortex immune stained for TNF*α* of: (a), (b), and (c), control, DLD, and DHD groups, respectively, showing negative immunoreactivity. (d) DOX treated group showing extensive expression in the renal glomeruli and renal tubules. (e) DOX/DLD group showing moderate expression within the glomeruli and the renal tubules. (f) DOX/DHD group showed marked improvement with no expression in glomeruli and renal tubules. The expression is mainly cytoplasmic, but with some immunopositive nuclei. Immunohistochemistry counter stained with H&E ×400. Bar = 20 *μ*.

Administration of DOX caused significant increase in the immunoreactivity of caspase-3, NF*κ*B, and TNF*α* (Figures 2, 3, and 4 and Table 4) respectively, which were highly expressed in both renal glomeruli and tubules cytoplasmically and in some nuclei. Administration of both doses of DIA concomitantly with DOX decreased the expression of them, compared to DOX group. Administration of both doses of DIA in vehicle treated rats alone and control groups showed no expression.

## 4. Discussion

Anticancer therapy usually demolishes the physiological homoeostasis and affects multiple organs during treatment process. Effective anticancer therapy with anthracyclines as DOX is limited because of its toxicity to various organs including kidneys [6]. Nephrotoxic action of DOX is also considered to be via drug-induced free radical generation [22]. The formation of free radicals induces the production of proinflammatory cytokines as interleukin 1 initiating the biological effects associated with inflammation [23]. This directed our attention to investigate the role of DIA which is interleukin 1 receptor antagonist as a possible nephroprotective agent against DOX-induced renal damage.

Induction of DOX nephrotoxicity was detected in our study by significant elevation of serum urea and creatinine levels which were confirmed by toxic histopathological changes compared to control group. Urea and serum creatinine are the most sensitive markers of nephrotoxicity implicated in the diagnosis of renal injury [24, 25]. The nephrotoxic effect of DOX is characterized by decreasing glomerular filtration rate leading to a rise in serum urea and creatinine. Our results are in good agreement with the previous studies [22, 26].

Improvement of DOX-induced nephrotoxicity was previously tried by compounds that partially succeeded in preserving normal renal function and structure probably through their antioxidant and anti-inflammatory effects as caffeic acid phenethyl ester [27],* Zingiber officinale* Roscoe [28], and* Solanum torvum* [26] so that we investigated the role of another antioxidant and anti-inflammatory drug as DIA on DOX-induced nephrotoxicity.

DIA could significantly decrease serum urea and creatinine compared to DOX treated group. That is due to the anti-inflammatory and antioxidant effects of DIA which suppress DOX mediated oxidative stress, inflammation, and tissue damage. Our histopathological changes showed that DOX treated group presented with marked damage of renal tubules. This is in agreement with Rashid et al. [22] and Al-Saedi et al. [29] who showed the same histopathological findings.

Coadministration of DIA significantly improved the histopathological changes compared to DOX treated group. These results are in agreement with Zhao et al. [30] who detected the protective effect of rhein (the active metabolite of DIA) on acetaminophen induced hepatotoxicity and nephrotoxicity in rats. They found that serum urea and creatinine significantly decreased in rhein and acetaminophen coadministration compared to acetaminophen group and normalization of toxic histopathological changes.

The elevated levels of GSH could effectively provide thiol group for the possible GSH mediated detoxification reactions of GPx (glutathione peroxidase) and GST (glutathione-s-transferase) which is involved in the scavenging of O_2_^−^ generated from the DOX [31]. Our findings are consistent with the previous reports that showed that GSH concentration is significantly decreased upon DOX treatment compared to control group [4, 22].

SOD extensively distributes in all cells and has a significant shielding role against oxidative injury induced by reactive oxygen species [22].

In our study, the activities of SOD and catalase significantly decreased in DOX treated rats in kidney as compared to control rats. The accumulation of these highly reactive free radicals leads to the reduction of the activity of SOD and catalase which in turn results in damaging effects in the form of loss of cell membrane integrity and function. The decrease in the SOD and catalase activities related to the increase in the intracellular levels of H_2_O_2_. Catalase has been reported to be responsible for the detoxification of H_2_O_2_, which is an effective inhibitor of SOD. Other researchers reported the same results [32, 33].

Coadministration of DIA significantly improved SOD, GSH, and catalase levels compared to DOX treated group. These results may be due to antioxidant effect of DIA which was approved previously by Tamura et al. [34] who indicated the inhibitory effect of DIA on indomethacin-induced gastric ulceration which could be mediated by the suppression of reactive oxygen species production based on its inhibition of neutrophil activation and antioxidant activity. In addition, Hu et al. [35] investigated the protective effects of rhein lysinate (RHL), against kidney impairment in senescence-prone inbred strain 10 (SAMP10) mice. Treatment of SAMP10 mice with RHL significantly increased the SOD and GPx levels in the kidneys.

Oxidative stress may damage cellular structures via lipid peroxidation of cellular membranes. O_2_^∙−^ reacts with lipid to form lipid peroxides followed by *β*-oxidation to form MDA [36]. That was detected in our study which showed significant increase of MDA level in DOX treated group compared to control group. These results are in agreement with El-Sheikh et al. [4] and Yagmurca et al. [27].

Another radical formatting mechanism in such an experimental protocol is NO_*x*_ producing system. The high production of NO_*x*_ results in peroxynitrite formation which is a potent and aggressive cellular oxidant and is involved in DOX toxicity [36]. The current findings showed that DOX administration significantly increased renal level of NO_*x*_ compared to control group and that is in agreement with other studies [26, 37].

Coadministration of DIA significantly decreased MDA and NO_*x*_ levels compared to DOX treated group. These results are in agreement with Zhao et al. [30] who detected the protective effect of rhein on acetaminophen induced nephrotoxicity in rats which was approved by significant decrease of MDA and NO_*x*_ on coadministration of rhein plus acetaminophen group compared to acetaminophen group. Our results are in agreement with Martel-Pelletier and Pelletier [38] who reported that NO is produced through the activity of inducible nitric oxide synthase and it is a major catabolic factor involved in the pathophysiology of OA. Interleukin 1*β* is a very potent stimulator of NO. Both DIA and rhein treatments markedly and significantly decreased interleukin 1*β*-induced NO production. Our results are consistent with Hu et al. [35] who investigated the protective effects of rhein lysinate (RHL), against kidney impairment in senescence-prone inbred strain 10 (SAMP10) mice. Treatment of SAMP10 mice with RHL significantly decreased MDA levels in the kidneys.

DOX treatment induced p53 phosphorylation. Induction of p53 mediates cell apoptosis through activation of caspase-3 family of proteases and apoptotic cell death [39]. Our study is showing significant increase in caspase-3 expression in DOX treated group in comparison with control group.

Coadministration of DIA significantly decreased caspase-3 expression compared to DOX treated group. Our study is in consistence with Torina et al. [40] who showed that treatment with DIA once a day for 4 weeks after myocardial infarction improved ventricular remodeling by partial blockage of the proinflammatory cytokines which led to lower caspase-3 activity and NF*κ*B p65 transcription B pathway.

DOX-induced superoxide anion production which was reported to be responsible for TNF*α*-induced nuclear factor (NF) activation that increases NF and TNF*α* over expression [41]. Our study showed significant increase in TNF*α* and NF*κ*B expressions in DOX group compared to control group and the same results were found with Al-Saedi et al. [29].

Coadministration of DIA significantly decreased TNF*α* and NF*κ*B expression compared to DOX treated group that is in agreement with Gadotti et al. [11] who showed that DIA inhibits neuropathic pain by decreasing proinflammatory cytokines as TNF*α* and NF*κβ*. Also, Hu et al. [42] hypothesized that the entity of diabetic nephropathy is inflammatory. The active metabolite of DIA is rhein which possesses anti-inflammatory activity and may be effective in suppressing the inflammatory cytokines contributing to the pathogenesis of diabetic nephropathy.

Moreover, Zhao et al. [30] demonstrated that rhein had protective effect in different models of nephropathy as IgA induced nephropathy, obstructive nephropathy, chronic allograft nephropathy, and high glucose and angiotensin II induced nephropathy. Oral administration of rhein (150 mg/kg/d) ameliorated renal lesions. Rhein was capable of protecting against renal injury by decreasing the activities of NF*κ*B and caspase-3 in the early phase of glomerulosclerosis [43].

Our results are consistent with Meng et al. [44] who reported that rhein possesses various pharmacological activities, including anti-inflammatory, antioxidant, and antitumor. In their study, a model of hyperuricemia and nephropathy induced by adenine and ethambutol in mice was established. The results demonstrated that rhein significantly improved the symptoms of nephropathy through decreasing the production of proinflammatory cytokines, including interleukin 1*β*, prostaglandin E2, and TNF*α*. Yu et al. [45] aimed to explore the effect of rhein on sepsis-induced acute kidney injury by injecting lipopolysaccharide (LPS) and cecal ligation and puncture (CLP) in vivo and on LPS-induced HK-2 cells in vitro. Rhein effectively attenuated the severity of renal injury. Rhein could significantly decrease concentration of serum urea and creatinine and level of TNF*α*, NF*κ*B, and IL-1*β* in two different mouse models of experimental sepsis.

## 5. Conclusion

In conclusion, DIA protected against DOX-induced nephrotoxicity in rats most probably due to its antioxidant and anti-inflammatory activities. However, DHD (50 mg/kg/day) showed more protective effect than DLD (25 mg/kg/day).

## Figures and Tables

**Table 1:** Effect of DLD (25 mg/kg/day) and DHD (50 mg/kg/day) on serum creatinine, serum urea, MDA, and NO_*x*_ levels in DOX -induced nephrotoxicity (15 mg/kg).

Group	Creatinine (mg/dL)	Urea (mg/dL)	MDA (nmol/g tissue)	NO_*x*_ (nmol/g tissue)
Control	0.8828 ± 0.07291	50.26 ± 4.450	42.27 ± 2.202	179.5 ± 11.98
DLD	0.7395 ± 0.06486	51.75 ± 1.721	44.77 ± 2.098	272.8 ± 22.35
DHD	0.9540 ± 0.08678	53.73 ± 2.989	52.02 ± 5.036	295.0 ± 20.25
DOX	1.475 ± 0.0522^a^	251.8 ± 12.23^a^	250.9 ± 16.37^a^	1114 ± 64.29^a^
DOX/DLD	1.211 ± 0.0660^ab^	241.7 ± 13.17^a^	51.86 ± 4.461^b^	322.8 ± 11.33^ab^
DOX/DHD	1.153 ± 0.0209^ab^	69.74 ± 4.161^b^	46.79 ± 1.39^b^	228.5 ± 18.83^b^

Values are representation of 4–6 observations as means ± SEM. Results are considered significantly different when *P* < 0.05. ^a^Significant difference compared to control; ^b^significant difference compared to DOX group.

**Table 2:** Effect of DLD (25 mg/kg/day) and DHD (50 mg/kg/day) on GSH, catalase, and SOD in DOX (15 mg/kg) induced nephrotoxicity.

Group	GSH (mmol/g tissue)	Catalase (unit/g tissue)	SOD (unit/g tissue)
Control	10.32 ± 0.2999	92.10 ± 2.835	829.7 ± 5.182
DLD	10.22 ± 0.5530	91.53 ± 1.860	832.0 ± 6.915
DHD	9.085 ± 0.3000	91.80 ± 2.127	826.6 ± 7.575
DOX	4.814 ± 0.1630^a^	72.49 ± 3.662^a^	657.6 ± 15.28^a^
DOX/DLD	8.678 ± 0.1985^ab^	80.48 ± 4.108	722.7 ± 41.13^a^
DOX/DHD	9.215 ± 0.2814^b^	8627 ± 4.496^b^	807.3 ± 16.96^b^

Values are representation of 4–6 observations as means ± SEM. Results are considered significantly different when *P* < 0.05. ^a^Significant difference compared to control; ^b^significant difference compared to DOX group.

**Table 3:** Scoring of morphological changes observed in control and experimental groups by light microscope (*n* = 6).

Findings	Control group	DLD group	DHD group	DOX treated group	DOX/DLD group	DOX/DHD group
(i) Glomerular vacuolations	−	−	−	++++	+	+
(ii) Enlarged renal corpuscles	−	−	−	++++	+	−
(iii) Tubular cells vacuolations	−	+	−	++++	+	+
(iv) Lumen widening	−	−	−	++++	+	−
(v) Distortion and Degeneration	−	−	−	++++	+	−
(vi) Casts	−	−	−	−	−	−

Animal groups tested are control untreated group, animals treated with diacerein (25 mg/kg/day, DLD) and diacerein (50 mg/kg/day, DHD), respectively, and animals treated with doxorubicin (DOX, 15 mg/kg), or with DOX together with low or high dose of diacerein (DOX/DLD or DOX/DHD), respectively.

Normal (−), in-between normal and mild (+), mild (++), moderate (+++), and severe (++++) [9].

**Table 4:** The effect of DLD and DHD doses on caspase-3, TNF*α*, and NF*κ*B immune expressions.

Group	Caspase-3	TNF*α*	NF*κ*B
Control	0.42 ± 0.80	0.40 ± 0.78	0.40 ± 0.88
DLD	0.60 ± 0.88	0.60 ± 0.80	0.60 ± 0.80
DHD	0.40 ± 0.40	2.40 ± 0.40	0.40 ± 0.40
DOX	58.60 ± 8.90^a^	80.60 ± 8.90^a^	58.60 ± 8.90^a^
DOX/DLD	30.20 ± 7.90^a/b^	35.20 ± 7.90^a/b^	25.20 ± 7.90^a/b^
DOX/DHD	10.00 ± 6.90^b^	5.00 ± 4.90^b^	10.00 ± 6.90^b^

Animal groups tested are control untreated group, animals treated with low or high doses of DIA alone (DLD or DHD), respectively, and animals treated with DOX or with DOX together with low or high dose of DIA (DOX/DLD or DOX/DHD), respectively.

^a^Significant from control group; ^b^significant from doxorubicin group.
