# Correction to “Possible Protective Effect of Diacerein on Doxorubicin‐Induced Nephrotoxicity in Rats”

**DOI:** 10.1155/jt/9868425

**Published:** 2026-06-26

M. M. M. Refaie, E. F. Amin, N. F. El‐Tahawy, and A. M. Abdelrahman, “Possible Protective Effect of Diacerein on Doxorubicin‐Induced Nephrotoxicity in Rats,” *Journal of Toxicology*, 2016, 9507563, https://doi.org/10.1155/2016/9507563.

In the article, there is an error in the figures, in which Figure 3b appears to be a zoomed‐out image of panel 4c, despite being described differently. The authors have confirmed that this was due to an error in figure preparation and provided the correct Figure 4 as below:

**Figure 4:**
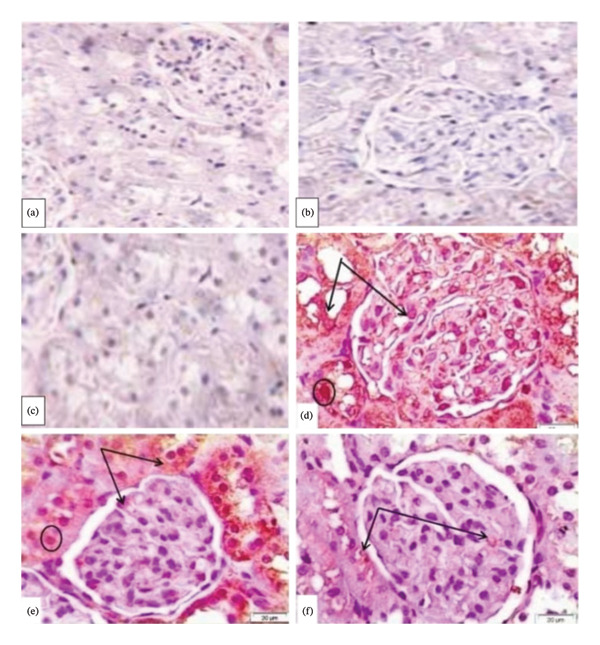
Photomicrographs of renal cortex immune stained for TNFα of (a), (b), and (c), control, DLD, and DHD groups, respectively, showing negative immunoreactivity. (d) DOX treated group showing extensive expression in the renal glomeruli and renal tubules. (e) DOX/DLD group showing moderate expression within the glomeruli and the renal tubules. (f) DOX/DHD group showed marked improvement with no expression in glomeruli and renal tubules. The expression is mainly cytoplasmic, but with some immunopositive nuclei. Immunohistochemistry counter stained with H&E × 400. Bar = 20µ.

We apologize for this error.

